# Efficacy of daily 800 IU vitamin D supplementation in reaching vitamin D sufficiency in nursing home residents: cross-sectional patient file study

**DOI:** 10.1186/1471-2318-14-103

**Published:** 2014-09-19

**Authors:** Bistra I Veleva, Victor G Chel, Wilco P Achterberg

**Affiliations:** Department of Public Health and Primary Care, Leiden University Medical Center, Leiden, The Netherlands; Nursing Home Topaz Overduin, Katwijk, The Netherlands

**Keywords:** Vitamin D sufficiency, Vitamin D supplementation, Nursing home, Cholecalciferol

## Abstract

**Background:**

The Dutch Health Council advises a standard daily vitamin D supplementation of 800 IU (20 mcg) for persons aged ≥ 70 years, with a target 25(OH)D serum concentration of ≥ 50 nmol/l. This recommendation is in line with advice from the Institute of Medicine (IOM) (2011) and the Expert Working Group on vitamin D (2012). A target 25(OH)D serum concentration of ≥ 75 nmol/l is also recommended in the literature. It is unknown whether this advice, initially designed for healthy adults/elderly, will lead to vitamin D sufficiency in the large majority of nursing home residents, taking into account the frailty of this population.

**Methods:**

Cross-sectional patient file study. Participants were 71 psychogeriatric nursing home residents (25 males, 46 females) with a mean age of 83 (SD 7) years using cholecalciferol capsules (5600 IU) once a week, or cholecalciferol drops (50,000 IU/ml) 3 drops a week (7500 IU), for at least 3 months. Main outcome measure was serum 25(OH)D level after supplementation.

**Results:**

Of all participants, 19 used cholecaliferol drops and 52 used cholecaliferol capsules. In total, mean serum 25(OH)D was 77 (SD 30) nmol/L and 55 residents (78%) were vitamin D sufficient. Among capsule users, mean serum 25(OH)D was 90 (SD 22) nmol/L and 49 (94%) were vitamin D sufficient. Among users of drops, mean serum 25(OH)D was 41 (SD 8) nmol/L and 6 (32%) were vitamin D sufficient.

**Conclusion:**

In most of these residents, vitamin D supplementation once a week with cholecalciferol capsules containing 5600 IU (equivalent to 800 IU daily) resulted in vitamin D sufficiency (serum 25(OH)D ≥ 50 nmol/L). When choosing a vitamin D preparation for routine supplementation in nursing home residents it should be noted that major differences may exist in efficacy, even when the various preparations contain the same amount of vitamin D.

## Background

Vitamin D deficiency (serum 25(OH)D < 30 nmol/L) and insufficiency (serum 25(OH)D > 30 < 50 nmol/L) [1] is common among older people as a result of reduction in mobility, time spent outdoors with sun exposure, intrinsic skin response to ultraviolet radiation and dietary vitamin D intake [2, 3]. In nursing home residents almost everyone is vitamin D insufficient if vitamin D is not supplemented [4, 5].

The importance of vitamin D (especially among older persons) is growing with increasing knowledge on the numerous biological effects of vitamin D as a promoter of bone health [6], physical performance [7] and as a possible modulator in, e.g., cardiovascular disease [8, 9], diabetes [10] and cancer [11]. In nursing homes, vitamin D supplementation is increasingly being considered as an indicator and standard for responsible care.

A consensus has not been reached yet among vitamin D researchers on the optimal 25(OH)D concentrations. The Endocrine Society clinical practice guidelines (2011) [12] recommend a target 25(OH)D serum concentration of ≥ 75 nmol/l with a daily vitamin D requirement of 1,500 -2,000 IU for persons aged ≥ 70 years.

The Dutch Health Council advises a standard daily vitamin D supplementation of 800 IU (20 mcg) for persons aged ≥ 70 years, with a target 25(OH)D serum concentration of ≥ 50 nmol/l [13]. This recommendation is in line with advice from the Institute of Medicine (IOM) (2011) [14] and the Expert Working Group on vitamin D (2012) [15].

However, it is unknown whether this advice, initially designed for healthy adults/elderly, will lead to vitamin D sufficiency in the large majority of nursing home residents, taking into account the frailty of this population with multiple comorbidities, polypharmacy, and dependency on basic activities of daily living. One study in Dutch nursing home residents, investigating the effect of equivalent oral doses of cholecalciferol 600 IU/day, 4200 IE/week and 18,000 IU/month on vitamin D status showed that, at 4 months, the percentage of patients with serum 25(OH)D < 50 nmol/L was 10.9% and 10.6% in the daily and weekly groups of vitamin D supplementation, respectively [4].

To our knowledge, no cross-sectional study in nursing home residents has investigated the efficacy of a daily vitamin D supplementation dose of 800 IU. Therefore, the present study investigates the prevalence of vitamin D sufficiency in a psychogeriatric nursing home population, after use of the recommended daily supplementation dose of 800 IU cholecalciferol.

## Methods

### Participants and intervention

A cross-sectional study was carried out in 71 residents of dementia care units of the nursing home *Topaz Overduin* in Katwijk (the Netherlands), using cholecalciferol capsules, once a week 5600 IE or cholecalciferol drops (50,000 IU/ml), 3 drops a week (7500 IU). The capsules contained cholecalciferol 100 IE/mg, cellulose microcrystalline PH102, magnesium stearate en lactose monohydrate (180). Drops were a watery mixture composed of cholecalciferol concentrate in oil, citric acid monohydrate, star anise oil, potassium sorbates, polysorbatum 80 (polyoxyethylene sorbitan monooleat), sugar syrup, and purified water.

The only exclusion criterion was the use of vitamin D for less than 3 months.

All blood samples were drawn on the same day.

The best parameter for vitamin D status, 25-hydroxyvitamin D [25(OH)D], was measured by a radioimmunoassay (25-OH-vitamin D RIA, Diasorin, Stillwater, MN, USA). The assay has 100% cross reactivity with 25(OH)D2 and 25(OH)D3. Total imprecision (Interassay coefficient of variation) is 9.4% at 22 nmol/l. SCAL Medical Diagnostics (Foundation central primary care laboratory) is certified by the Dutch Board for Accreditation and participates in external quality assessment schemes organized by the Foundation Quality Control Medical Laboratory Diagnostics.

### Ethical approval

Ethical approval was not necessary under Dutch regulations since this study was a retrospective patient file study. Among all elderly care physicians in the participating nursing home, drawing blood samples, in order to check the effects of vitamin D supplementation on individual serum 25(OH)D levels, is considered to be a quality of care standard and therefore part of good medical practice. Therefore, the drawing of blood samples was for clinical purposes and complied with The Dutch Law of Agreement to Medical Treatment (WGBO), and not as research that has to comply with the Dutch law on Medical Research in Humans (WMO), for which ethical approval is required. Informed consent however is also necessary for all diagnostic and clinical work under the WGBO, so patients who did not want their blood samples taken, could refuse.

### Potential factors that influence *25(OH)D concentration*

From the patient files, data were collected on factors possibly influencing a rise in serum 25(OH)D: age, comorbidity, number and sort of medication use (anticonvulsant medication and corticosteroids were taken into account because these increase catabolism of 25(OH)D [13]), body mass index (BMI), sun exposure, Modification of Diet in Renal Disease (MDRD) as an estimate of the renal function, and Functional Ambulation Classification (FAC) scores as an assessment of the mobility, performed by physiotherapists and physicians. The FAC consists of five items. Scores range from 0–5 (0 = Nonfunctional ambulation; 1 = Ambulator-dependent for physical assistance level II; 2 = Ambulator-dependent for physical assistance level I; 3 = Ambulator-dependent for supervision; 4 = Ambulator- independent level surfaces only; and 5 = Ambulator independent) [16].

Comorbidity was expressed as the number of chronic diseases, taking into account 7 major conditions [17], i.e. chronic obstructive pulmonary disease, cardiac disease, peripheral arterial disease, diabetes mellitus, stroke, cancer and rheumatoid arthritis/osteoarthritis, and measured by review of the patients’ medical files. BMI was calculated as body weight in kg divided by height in m^2^ and subsequently categorized into three groups: underweight (BMI < 20 kg/m^2^), normal weight (BMI: 20-25kg/m^2^) and overweight (BMI > 25 kg/m^2^).

Adequate compliance was defined to exist when more than 80% of the vitamin D medication was ingested.

### Statistical analysis

Main outcome measure was serum 25(OH)D level after supplementation.

We express continuous variables as means ± standard deviation and categorical variables as percentages. We used Student’s T for comparisons, as well as Chi-squared test.

A logistic regression analysis was used to assess the possible predictors of 25(OH)D insufficiency. P-values were considered significant at a p-value < 0.05. Odds ratios (OR) and 95% confidence intervals (95% CI) were calculated to estimate the strength of the association when the p-value was significant. All analyses were conducted with SPSS 21 software.

## Results

Of the 71 participants with at least 3 months supplementation, 19 used cholecaliferol drops (3 males/16 females) and 52 used cholecaliferol capsules (14 males/38 females). In total, mean serum 25(OH)D was 77 (SD 30) nmol/L and 55 residents (78%) were vitamin D sufficient (Table 1).

**Table 1:** **Characteristics of 71 patients with vitamin D supplementation**

	Total group	Supplementation by capsules	Supplementation by drops	P
Subjects (females/males), n	71 (46/25)	52 (38/14)	19 (16/3)	0.5
Age in years, mean (SD)	83 (7)	83 (7)	82 (8)	0.4
Serum 25(OH)D^1^ nmol/L, mean (SD)	77 (30)	90 (22)	41 (18)	<0.001
Ser. 25(OH)D in residents, % (n)				
≥75	61 (43)	80 (42)	5 (1)	
≥50-74.9	17 (12)	14 (7)	26 (5)	
30-49.9 nmol/L	14 (10)	6 (3)	37 (7)	
< 30 nmol/L	8 (6)	0 (0)	32 (6)	
Duration supplementation% (n)				<0.001
3-6 months	24 (17)	33 (17)	0 (0)	
12-18 months	45 (32)	60 (31)	5 (1)	
>18 months	31 (22)	7 (4)	95 (18)	
Subjects with sunlight exposure >1 × week% (n)	61 (43)	44 (23)	74 (14)	0.2
Number of medications% (n)				0.9
<5	73 (52)	73 (38)	74 (14)	
>5	27 (19)	27 (14)	26 (5)	
Medication influencing 25(OH)D	15 (11)	15 (8)	15 (3)	0.8
- anticonvulsants	1 (1)	2 (1)	0	
- corticosteroids	14 (10)	13 (7)	15 (3)	
FAC^2^, % (n)				0.3
0	39 (27)	43 (23)	21 (4)	
1	11 (8)	10 (5)	16 (3)	
2	11 (8)	10 (5)	16 (3)	
3	14 (10)	10 (5)	26 (5)	
4	21 (15)	23 (12)	16 (3)	
5	4 (3)	4 (2)	5 (1)	
BMI^3^, % (n)				0.01
<20 underweight	55 (39)	60 (31)	42 (8)	
20-25 healthy weight	34 (24)	34 (18)	32 (6)	
>25 overweight	11 (8)	6 (3)	26 (5)	
MDRD^4^, % (n)				0.7
<60	31 (22)	33 (17)	26 (5)	
>60	69 (49)	67 (35)	74 (14)	
Chronic disease^5^, % (n)				0.6
≤2	73 (52)	73 (38)	74 (14)	
>2	27 (19)	27 (14)	26 (5)	

Values are mean (SD) or number (percentage).

Of the 52 capsule users, mean age was 83 (SD 7) years and mean serum 25(OH)D was 90 (SD 22) nmol/L. None of this group was vitamin D deficient [25(OH)D < 30 nmol/L], whereas 3 (6%) were vitamin D insufficient [25(OH)D <50 nmol/L], 49 (94%) were vitamin D sufficient [25(OH)D ≥ 50 nmol/L] and 42 of this group (80%) had serum 25(OH)D levels > 75 nmol/L.

Of the 19 drop users, mean age was 82 (SD 8) years and mean serum 25(OH)D level was 41 (SD 18) nmol/L. In this group, 6 (32%) were vitamin D deficient [25(OH)D < 30 nmol/L], 7 (37%) were vitamin D insufficient [25(OH)D <50 nmol/L], 6 (31%) were vitamin D sufficient [25(OH)D ≥50 nmol/L], and 1 (5%) had serum 25(OH)D levels > 75 nmol/L.

Among drops user, all 19 (100%) participants were compliant. Among capsule users 50 (96%) were compliant and 2 (4%) were not.

Because of the lower serum concentration of 25(OH)D in the group of drop users we used Student’s T for comparisons as well as Chi-squared test to compare the basic characteristics of the two groups that can influence the 25(OH)D concentration. A significant difference was established in the BMI between the two groups (p 0.01) with a higher number of persons with overweight in the group of the drop users. Subsequently we carried out a bivariate analysis with the serum vitamin D concentration (patients divided in two groups 25(OH)D < 50 nmol/l and 25(OH)D ≥ 50 nmol/l) as dependent variable to control for potential confounders that can influence vitamin D concentration.

The use of cholecalciferol drops was a strong predictor of 25(OH)D insufficiency (OR 35.3; p < 0.0001; 95% CI 7.7-160.9) (Table 2).

**Table 2:** **Predictors of vitamin D insufficiency in patients with dementia with Vitamin D supplementation: binary logistic regression analyses**

	25(OH)D^1^ < 50 nmol/L	25(OH)D ≥50 nmol/L	P-value	OR	95% CI
N	16	55			
Gender, % (n)			0.9		
Male	25 (12)	23 (13)			
Female	75 (4)	76 (42)			
Age in years, mean (SD)	83 (SD 8)	83 (SD 7)	0.9		
Subjects with sunlight exposure >1x/week, % (n)	25 (4)	44 (24)	0.1		
Drops/capsules, % (n)			<0.0001	35.3	7.7-160.9
- drop users	81 (13)	11 (6)			
- capsule users	19 (3)	89 (49)			
No. of medications, % (n)			0.8		
<5	75 (12)	72 (40)			
>5	25 (4)	27 (15)			
FAC^2^, % (n)			0.3		
0	25 (4)	42 (23)			
1	6 (1)	13 (7)			
2	19 (3)	9 (5)			
3	31 (5)	9 (5)			
4	13 (2)	24 (13)			
5	6 (1)	4 (2)			
BMI^3^, (n)			0.3		
<20 underweight	56 (9)	55 (30)			
20-25 healthy weight	25 (4)	36 (20)			
>25 overweight	19 (3)	9 (5)			
MDRD^4^, % (n)			0.6		
<60	37 (6)	29 (16)			
>60	62 (10)	71 (39)			
Comorbidity^5^, % (n)			0.02	0.2	0.07-0.8
≤ 2 diseases	50 (8)	80 (44)			
>2 diseases	50 (8)	20 (11)			

^1^25(OH)D −25-hydroxyvitamin D.

^2^FAC - Functional Ambulation Classification.

^3^BMI - Body Mass Index.

^4^MDRD - Modification of Diet in Renal Disease.

^5^Chronic diseases from seven majors: chronic obstructive pulmonary disease, cardiac disease, peripheral arterial disease, stroke, diabetes mellitus, rheumatoid arthritis/osteoarthritis and cancer.

In the total study population, residents with 25(OH)D serum levels ≥50 nmol/L were more likely to have less comorbidity: i.e. ≤ 2 chronic somatic diseases registered (p = 0.02; OR 4.0; CI 1.2-13.0). However, this correlation was not significant among drop users. Among capsule users, the number of vitamin D insufficient subjects was too low (<5) to examine this correlation.

In both groups, no association was found between the other possible confounders (gender, age, BMI, renal function, sun exposure, number and kind of medication and mobility status) and vitamin D insufficiency.

## Discussion

Vitamin D supplementation that may be needed to achieve optimal concentration of 25(OH)D in all populations is not established. Studies suggest that 700 to 1000 IU vitamin D per day may be enough to bring 50% of the younger and older adults up to 75–100 nmol/l [18–20]. In our study a supplementation of 5600 IU vitamin D once a week by capsules brings 80% of the older adults to serum 25(OH)D higher than 75 nmol/l.

A strength of the present study is that it is the first to demonstrate that a population-based approach in vitamin D supplementation strategy is feasible, even in a population of fragile institutionalized older people.

However, because it is a single-site study with a relatively small number of patients the external validity is limited. The study was conducted in Dutch nursing home residents and recommendations are for this population. Reproduction of the study in multiple sites and countries is therefore warranted.

The present study reveals a striking difference between the efficacy of drops and capsules in reaching vitamin D sufficiency in a psychogeriatric nursing home population.

In capsules users only 3 of 52 subjects (6%) were vitamin D insufficient; 2 of these participants had low medication compliance because of unwillingness to take the medication (presumably related to their cognitive decline). The third insufficient resident had a history of recurrent bladder carcinoma, long carcinoma and radiotherapy, cardiac disease and chronic renal failure. Among users of drops, no less than 13 of 19 subjects (69%) had insufficient 25(OH) serum levels. Possible reasons for this discrepancy were further investigated.

Indeed we didn’t have the baseline 25(OH)D levels of our patients before the supplementation. In a previous study conducted in Netherlands, vitamin D baseline level was found to be insufficient in 98% of the nursing home residents [4]. It is also obvious in our study that the drop users have a longer duration of vitamin D supplementation than capsule users (p < 0.0001) and no one of the drop users receives the supplementation shorter than one year.

Compliance with the ingestion of drops was 100% in all participants; drops were administered by spoon with apple sauce, or in a small quantity of tea or water. In no case did drops exceeded the maximum shelf life and all were stored as specified by the pharmacist; the pharmacist also confirmed that 3 drops do in fact contain 7500 IU of cholecalciferol. Drops were a watery mixture composed of cholecalciferol concentrate in oil, citric acid monohydrate, star anise oil, potassium sorbates, polysorbatum 80 (polyoxyethylene sorbitan monooleat), sugar syrup, and purified water, which appears to have better bioavailability than oily formulations [21]. In the nursing home *Topaz Overduin* the method used for supplementing vitamin D has changed over time from drops to capsules; this means that there was no specific reason why any resident should still be using drops.

The nursing staff was asked about the way drops were administered. They reported this occurred in 3 ways, depending on the nurse’s personal choice: 1) 3 drops were administered directly from the drop container, 2) 3 drops were given using a 1-ml syringe (delivered standard by the pharmacist with the drop container and the instruction that 0.11 ml be taken in case of syringe use), or 3) 0.11 ml was administrated with the 1-ml syringe. Over time, each resident received the drops in these different ways, implying that there was no set procedure of administration for any particular group.

A literature search for confounders in attaining accuracy and precision of delivery from containers with oral drops, yielded several pediatric and ophthalmologic studies [22–24]. Interestingly one of these studies, assessing the dose uniformity of samples delivered from pediatric oral droppers, discovered that the key factor in achieving satisfactory dispensing is the position of the dropper - which has to be held vertically [22].

In our nursing home, the two other routes of administration of drops are also susceptible to dosage errors, i.e. accurate titration of 0.11 ml cholecalciferol with a 1-ml syringe is practically impossible; also, drops given via a syringe have a different volume than drops given with the container. Since the nursing staff of our nursing home received no instructions concerning the administration of drops, this might be an explanation for the discrepancy found. Because dosage errors are possible when administering small volumes of solutions, nursing staff should be guided on the correct method of delivery.

## Conclusion

Vitamin D supplementation using cholecalciferol capsules containing 5600 IU, once a week (equal to 800 IU daily) will result in vitamin D sufficiency (serum 25(OH)D ≥ 50 nmol/L), regardless of gender, age, BMI, renal function, sun exposure, comorbidity, medication and mobility status. When choosing a vitamin D preparation for routine supplementation for nursing home residents, it is important to note that major differences in efficacy may exist between various types of preparations, even when they apparently contain the same amount of vitamin D. This raises the issue of standardization of drops administration for the purpose of avoiding failure in the meeting of recommended daily needs of vitamin D.

## Authors’ information

Wilco P. Achterberg-professor of institutional care and elderly care medicine, Leiden University Medical Center, Department of Public Health and Primary Care, specialties: geriatrics, nursing home medicine, pain, dementia, geriatric rehabilitation.

Victor G. Chel, elderly care physician, completed his PhD on “Treatment of vitamin D deficiency in Dutch nursing home residents”. Head of the specialist training programme for elderly care physicians, Leiden University Medical Center, Department of Public Health and Primary Care,

Bistra I. Veleva- a physician in training at Leiden University Medical Center, Department of Public Health and Primary Care, specialist training programme for elderly care physicians.

Bistra Veleva and Wilco Achterberg are alternate corresponding authors.

## Abbreviations

IOM: Institute of Medicine
25(OH)D: 25-hydroxyvitamin D
FAC: Functional Ambulation Classification
BMI: Body Mass Index
MDRD: Modification of Diet in Renal Disease
Chronic diseases: chronic obstructive pulmonary disease, cardiac disease, peripheral arterial disease, stroke, diabetes mellitus, rheumatoid arthritis/osteoarthritis and cancer.
